# A Tremendous Reorganization Journey for the 3D Chromatin Structure from Gametes to Embryos

**DOI:** 10.3390/genes13101864

**Published:** 2022-10-15

**Authors:** Zhenping Chen, Xuepeng Chen

**Affiliations:** 1 Bioland Laboratory (Guangzhou Regenerative Medicine and Health Guangdong Laboratory), Guangzhou 510320, China; 2 Guangzhou Laboratory, Guangzhou 510320, China

**Keywords:** 3D chromatin structure, gametes, zygotes, ZGA, chromatin structure reorganization

## Abstract

The 3D chromatin structure within the nucleus is important for gene expression regulation and correct developmental programs. Recently, the rapid development of low-input chromatin conformation capture technologies has made it possible to study 3D chromatin structures in gametes, zygotes and early embryos in a variety of species, including flies, vertebrates and mammals. There are distinct 3D chromatin structures within the male and female gametes. Following the fertilization of male and female gametes, fertilized eggs undergo drastic epigenetic reprogramming at multi levels, including the 3D chromatin structure, to convert the terminally differentiated gamete state into the totipotent state, which can give rise to an individual. However, to what extent the 3D chromatin structure reorganization is evolutionarily conserved and what the underlying mechanisms are for the tremendous reorganization in early embryos remain elusive. Here, we review the latest findings on the 3D chromatin structure reorganization during embryogenesis, and discuss the convergent and divergent reprogramming patterns and key molecular mechanisms for the 3D chromatin structure reorganization from gametes to embryos in different species. These findings shed light on how the 3D chromatin structure reorganization contribute to embryo development in different species. The findings also indicate the role of the 3D chromatin structure on the acquisition of totipotent developmental potential.

## 1. Introduction 

In eukaryotes, the vast majority of genetic information is stored, transcribed and replicated within the nucleus. The 2-meter-long genomic DNA must be compacted in order to be accommodated into the nucleus, which can range from 2–10 μm in diameter. Besides this challenging topological problem, the organization of the genome must enable the gene expression program to be executed at the right time and in the right cell types [1]. The chromatin within the interphase nucleus is organized in a hierarchical manner. Chromosomes are not randomly distributed within the nucleus but instead occupy distinct territories [2]. Then, the chromosomes are thought to be divided into A/B compartments at a multi-megabase scale. ‘A’ compartments prefer to occupy the internal section of the nucleus and typically contain active genes, while ‘B’ compartments occupy the periphery of the nucleus containing inactive genes [3,4]. Furthermore, at the secondary sub-megabase level, the chromatin is organized into hundreds of self-associating domains which are typically termed as topologically associated domains (TADs) [5,6]. A TAD is a continuous chromatin segment. Interactions between genomic regulatory elements and genes are more frequent inside a TAD than between two different TADs [5,6]. TAD segmentations are quite stable in different cell types [7,8,9,10], which are regarded as the basic unit of the folded genome [11,12,13].

In the eukaryote, the development of embryos starts with fertilization, namely the fusion of sperm and oocytes. During the embryogenesis following the fertilization process, one of the most important developmental events is the maternal to zygotic transition (MZT). The embryonic development program is initially controlled by the maternal deposited factors. Then, the zygotic genome activation (ZGA) starts, and the embryonic developmental program gradually becomes under the control of zygotic genome factors. The ZGA timing varies widely in different organisms, from the 14th mitotic division stage in drosophila which is approximately 2.5 h post-fertilization (2.5 hpf) to the 8-cell stage in humans which is approximately 3 days post-fertilization (3 dpf) [14,15,16]. During the MZT, maternal deposited factors are largely degraded and replaced by newly transcribed embryonic factors. These embryonic factors have pivotal roles in developmental regulation [17]. In addition to the genome-wide activation of transcription, the zygotic genome undergoes drastic epigenetic reprogramming [17,18]. With the help of low-input chromatin conformation capture methods and imaging technologies, recent studies have revealed surprising characteristics of the chromatin structures in early embryos [8,19,20,21,22,23,24,25,26]. The chromatin structure characteristics in early embryos include the immediate and significant relaxation of the chromatin structure after fertilization, which is very distinct from the other somatic cells. How does the three-dimensional chromatin structure undergo such a drastic reorganization from gametes to embryo? What mechanisms are employed for the chromatin structure reorganization in the embryos of different species? 

Here, we review the recent findings for the 3D genome reorganization from gametes to embryos in different species and discuss the mechanisms and the conservation of the 3D genome reorganization across different species.

## 2. The Chromatin Organization in Male and Female Gametes

### 2.1. The Chromatin Organization in Sperm

Prior to fertilization, the maternal and paternal gametes are terminally differentiated cells [27]. The chromatin composition in mature sperm is significantly different from that in somatic cells. In drosophila and mammals, the vast majority of the sperm genome is packaged by protamine [28,29,30]. Only a small proportion of histones remain focally at the many promoters and enhancers of housekeeping and developmental genes in both mouse and human embryos [31,32]. In contrast to most vertebrate species, zebrafish sperm are packaged entirely by histones rather than protamine proteins [33,34,35].

**Figure 1:**
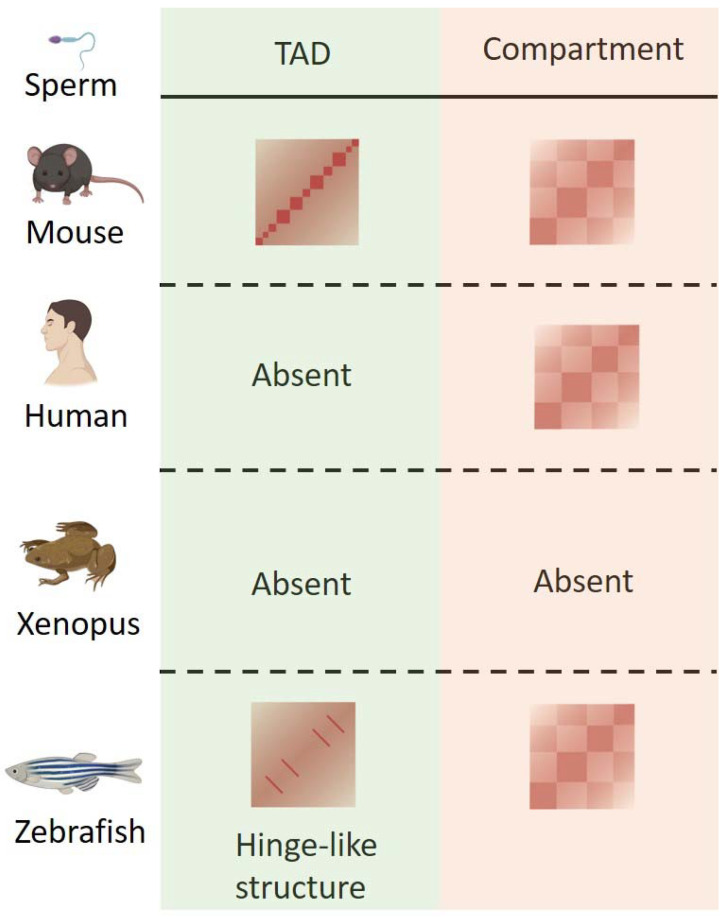
TADs and compartment structures in sperms of different species. TADs are present in mouse and zebrafish sperm, but are absent in human and xenopus sperm. Mouse sperm has canonical TAD structures. Zebrafish sperm shows unique “hinge-like” TAD structures. As for A/B compartments, clear A/B compartment structures can be detected in mouse, human and zebrafish sperm but not in xenopus sperm.

Mature sperm are typically haploid and transcriptionally inactive, even though a small number of promoters appear to retain nucleosome-free regions with several core transcription factors [36]. The chromatin organization in mature sperm is quite different among species (Figure 1). In mice, the clear TADs and A/B compartment structures are stored in mature sperm [23,26,36,37,38]. By contrast, human sperm has clear A/B compartments but does not have TADs [22]. The loss of TADs in human sperm is most likely due to the absence of the CTCF protein [22]. The spermatogenesis process in drosophila is similar to that in mammals [39]. However, as far as we know, there are no sequencing data to decipher the real TADs and A/B compartments in mature drosophila sperm. Furthermore, Niu et al. found that neither TADs nor compartments can be detected in xenopus sperm [21]. Surprisingly, the zebrafish sperm chromatin, which is totally packaged by histones, lacks canonical TADs [19]. Instead, the chromatin in zebrafish sperm displays “hinge-like” domain structures with an average 150 kb in size and repeat per 1–2 Mb [19]. The chromatin structure in zebrafish sperm is similar to the mitotic flower spiral structure that has been proposed for mitotic chromosomes [40,41]. However, it is still unknown why mature sperm in different species display such divergent chromatin organization. The mechanisms and biological significances for the divergent sperm chromatin organization need to be further explored.

### 2.2. The Chromatin Organization in Oocytes 

For the chromatin organization in oocytes, Flyamer et al. reveals a global interaction shift during the transition from transcriptionally active immature oocytes to transcriptionally silent mature oocytes in mouse [8]. Using the aggregate analysis for loops and TADs, Flyamer et al. show that the intensity of TADs and loops significantly decrease during the oocyte maturation [8]. Two other independent studies examined mature mouse oocytes at the MII stage, and did not detect any TADs [23,26]. Altogether, these results show that in mice, the TAD and loop strength decrease progressively during the oocyte maturation and that there are no TADs in mature oocytes due to the metaphase II phase.

## 3. The Chromatin Structure Reorganization during Embryogenesis

**Figure 2:**
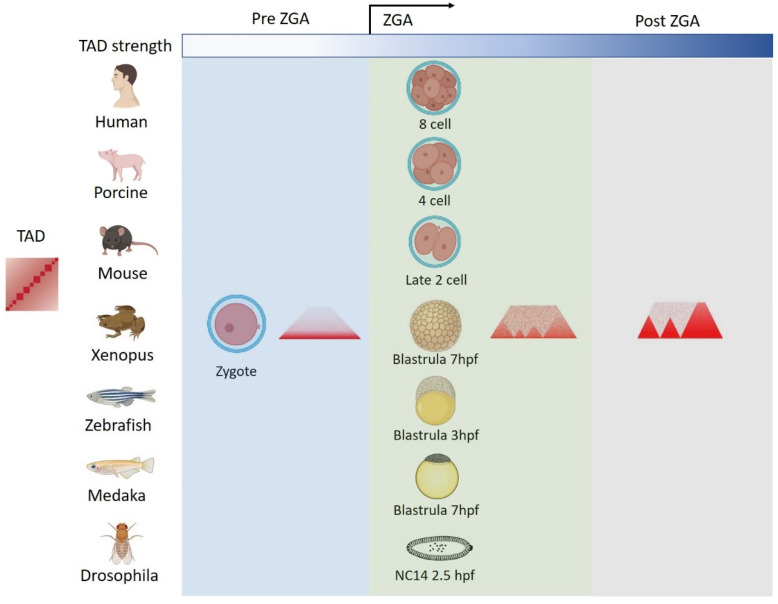
TAD reprogramming during the early development in different species. A schematic showing the TAD reprogramming during embryogenesis in human, porcine, mouse, zebrafish, medaka, xenopus and drosophila embryos. Overall, the establishment of TADs is coincident with the ZGA time windows in these studied organisms. Briefly, after fertilization, the TAD structures are largely lost in pre-ZGA embryos and then gradually established in post-ZGA embryos.

After the fertilization process in animals, two mature parental gametes fuse and produce a fertilized egg. At first, the fertilized egg is transcriptionally inactive [42], and then zygotic genome activation (ZGA) starts. The ZGA is characterized by the widespread recruitment of RNA polymerase II (RNA Pol II) [43,44,45] and genome-wide transcriptional activation, accompanied by a genome accessibility increase [46,47,48] and histone modification changes [34,49,50,51]. Furthermore, recent studies have also revealed the drastic reprogramming of higher-order chromatin structures from gametes to early embryos [8,19,20,21,22,23,24,25,26,52,53,54,55,56] (Figure 2). The results indicate that TADs reestablish during embryogenesis, and the timing of TAD reestablishment coincides with the ZGA time window in many species (Figure 2). The earliest studies of 3D genome organization in embryos were carried out in mouse and drosophila embryos [23,25,26]. In mice, the ZGA occurs at the late 2-cell stage. TAD structures emerge at the late 2-cell stage, and then gradually become consolidated and mature by the 8-cell stage in mice [23,26]. In drosophila embryos, only the nucleus is divided with no cytoplasm division at first. Once the nucleus has reached the 13th cycle of division (NC13), the cell membrane is formed. At the NC14, the ZGA stage of drosophila, TADs then begin to establish [25]. Subsequently, similar TAD reestablishment processes are also observed in human [22], porcine [54], medaka [20], and xenopus embryos [21]. In human and porcine embryos, TADs appear at ZGA (the 8-cell stage in human or 4-cell stage in porcine) and become increasingly evident during the embryonic development [22,54]. A similar pattern is also seen in medaka and xenopus embryos, in both of which ZGA occurs at 7hpf [20,21]. However, in zebrafish, one earlier study suggested that TADs were present before ZGA but dissolved at the ZGA, and then reestablished at the later stage after ZGA [57]. By contrast, recently Wike et al. revealed a contrary result and suggested that the presence of TADs before ZGA in zebrafish embryos appears to be a contaminating artifact of somatic cells [19]. Wike et al. further showed that TADs start to establish in the post-ZGA embryos, not in the pre-ZGA embryos, and that TAD boundaries become stronger and more numerous during the zebrafish development [19].

**Figure 3:**
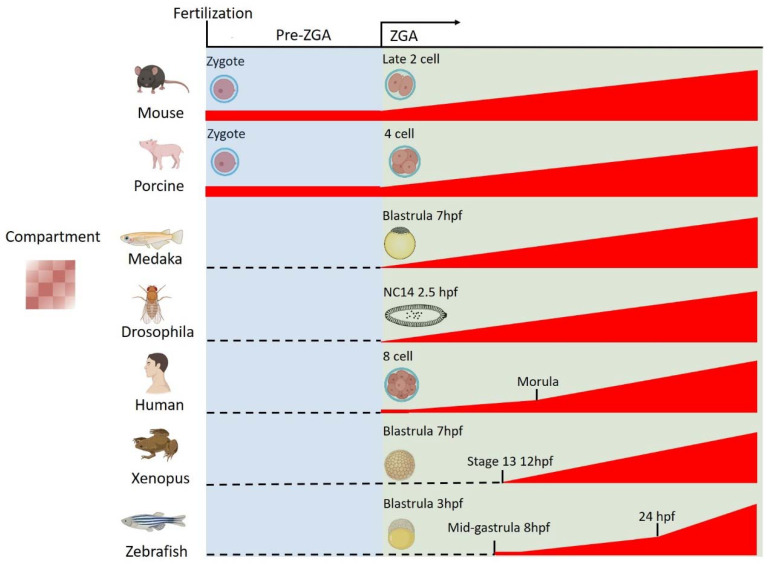
A/B compartment reprogramming during the early development in different species. The emergence timing of A/B compartments in early embryos is highly species-specific. In mouse and porcine embryos, A/B compartments already exist before ZGA and are gradually enhanced after ZGA. In medaka and drosophila, A/B compartments begin to establish at ZGA. In human, only weak A/B compartments exist at ZGA and become stronger from the morula stage onwards. By contrast, the establishment of A/B compartments mainly occurs after ZGA in xenopus and zebrafish embryos.

Interestingly, while the establishment of TAD and TAD boundaries is coincident with the ZGA time windows in these studied organisms, the emergence timing of A/B compartments is much more variable (Figure 3). For the A/B compartmentalization in mice, Ke et al. and Du et al. report that A/B compartmentalization already exists before ZGA and gradually enhances after ZGA [23,26]. Similarly, the gradual A/B compartmentalization could also be observed in porcine embryos, and chromosomal segregation increased during porcine embryogenesis especially from 4-cell stage to the morula [54]. In drosophila and medaka, A/B compartmentalization cannot be detected before ZGA and begins to form at ZGA [20,25]. In xenopus embryos, the appearance of A/B compartments starts as early as the 13th stage, shortly after ZGA [21]. In humans, A/B compartmentalization is reported to be absent at the 2-cell stage and remains weak even at the ZGA around the 8-cell stage, but then clear compartmentalization can be detected at the morula and blastocyst stages [22]. By contrast, in zebrafish, compartments are absent before mid-gastrula (8 hpf), and strong compartmentalization is observed at the late segmentation stage (24 hpf) [19,20]. 

Taken together, these results show a drastic reorganization of 3D chromatin structure during embryogenesis in different species although the emergence timing varies considerably. 

## 4. Mechanisms of 3D Chromatin Structure Reorganization in Embryos

An important question in the chromatin biology field is how the structural features of 3D chromatin organization are established, maintained and potentially reset during the cell cycle, development and stimulus signaling. Different species seem to deploy different components to establish chromatin domains [58]. Here, we review the recent findings of different mechanisms for chromatin organization at ZGA.

### 4.1. Zygotic Genome Activation

Given the coincidence of TAD emergence and ZGA, some are wondering whether ZGA can facilitate the de novo establishment of the 3D genome in early embryos. Previous reports show that TAD establishment is independent of ZGA in mouse and drosophila embryos [23,25,26]. In these studies, it was found that chromatin can still establish TADs after the inhibition of ZGA by *α-amanitin*, indicating that the establishment of a TAD structure does not depend on ZGA in mouse and drosophila embryos. Strikingly, a study in human early embryos reveals that TAD establishment in human embryos requires ZGA. TAD structures become much more obscure in *α-amanitin*-treated 8-cell embryos than that in untreated 8-cell embryos, suggesting that ZGA plays a role in promoting TADs in human embryos [22]. The gradual establishment of a 3D chromatin structure is accompanied by the occurrence of ZGA but does not rely on the ZGA in mice. 

### 4.2. DNA Replication

According to recent studies, 3D genome organization at different scales are reported to be related to DNA replication. During the cell cycle, the A compartments prefer to replicate at the early stage, whereas B compartments prefer to replicate at the late stage [59,60,61]. DNA replication domains typically overlap with TADs in mammalian cells [62]. TAD structures can undergo reprogramming during the mitotic cell cycle [40]. Clear TAD structures can be detected at G1 and S phases but not at the metaphase [40]. To investigate the role of DNA replication in regulating the TAD domain establishment, Ke et al. treated mouse 2-cell embryos with aphidicolin inhibitor to block the DNA replication. The results show that the early embryos cannot establish TAD structures, indicating that the establishment of the TAD structure depends on the DNA replication at the 2-cell stage in mice [26].

### 4.3. Insulator Proteins

The loop-extrusion model was proposed to explain the formation mechanism of TAD domains. TAD boundaries are often enriched for insulator proteins which can block interactions between neighboring genomic regions. The CCCTC-binding factor (CTCF), which is originally characterized as an insulator protein, is capable of restricting enhancer–promoter interactions both in reporter plasmids and in their native environment [63,64]. CTCF contains an 11-zinc-finger DNA-binding domain which can recognize a specific non-palindromic motif [65]. CTCF is highly conserved in most bilaterians [65,66] and is essential for embryonic development [43,66]. In mammals and zebrafish, most TAD boundaries are enriched for the insulator protein CTCF along with cohesin [67]. In the mouse embryonic development, the depletion of maternal deposited and zygotic CTCF leads to embryonic lethality [68,69]. At the TAD organization level, CTCF-depleted mouse blastocysts showed a reduction in the number of TADs across the genome, leading to an increase in median size, and a high degree of TAD reorganization [69]. In humans, most of the CTCF is not maternally inherited, and although the CTCF expression at the ZGA is required, it is not sufficient for TAD formation in human embryos [22]. CTCF is also required for correct TAD formation in zebrafish [70] and in xenopus embryos [21]. However, in drosophila embryos, dCTCF does not play a major role in domain formation [71], and the dCTCF knockout only affects a small proportion of domain boundaries [72]. While CTCF is required for proper embryonic development, the dependence of the 3D chromatin structure reorganization in embryos on CTCF still needs to be further investigated in other species.

Besides CTCF, the cohesin complex, which mainly consists of SMCs (structural maintenance of chromosomes proteins), can physically bind replicated DNA and play an important role in sister chromatid cohesion during mitosis [73,74,75,76,77]. Recently, it has also been found that cohesin can play functions in TAD formation. Cohesin can form a ring structure and function as a driver to extrude a chromatin loop, and then be blocked by a pair of convergent CTCF sites to form TAD boundaries [67,78,79]. In mammalian somatic cells, the removal of cohesin loading on the chromatin can obviously disrupt the TAD domains [80,81]. Similarly, in mouse embryonic stem cells, the cohesin loss can also eliminate TADs and reinforce A/B compartmentalization [82]. 

In addition, other architectural proteins such as BEAF-32, Cp190, and Chromator are proposed to involve in TAD boundary formation by mediating long range chromosol contacts in drosophila [83,84] (Table 1).

### 4.4. Non-Insulator Proteins

Besides insulator proteins, several non-insulator proteins are also reported to play important roles in the regulation of 3D chromatin structure reorganization in embryos. In drosophila, transcription factors have been identified to be involved in the establishment of chromatin organization at the ZGA, such as Zelda. Zelda is an essential pioneer transcription factor which activates hundreds of genes throughout ZGA and is required for opening chromatin accessibility in drosophila [88]. TAD boundaries that appear during drosophila ZGA need to be bound by Zelda [25]. Additionally, the formation of multi-way interaction hubs between enhancers and promoters requires Zelda [56]. Moreover, according to the co-occupancy in the genome, GAF may function together with Zelda to determine regions of open chromatin and regulate domain formation [86]. Recently, a study revealed that heterochromatin protein 1α (HP1α) is also essential for de novo 3D genome establishment in drosophila embryos [87]. Decreased HP1α binding in peri-centromeric heterochromatin can lead to a declustering and decondensation of the constitutive heterochromatin and a perturbed Rabl configuration [87]. Meanwhile, HP1α also binds genomic regions where they are enriched for H3K9me3 modifications and repeats. Such binding of HP1α is involved in regulating chromatin folding and the formation of B compartments. The depletion of HP1α specifically affects B compartments but not A compartments [87]. Interestingly, the depletion of HP1α only has a little effect on ZGA [87]. Furthermore, HP1α depletion also does not affect chromatin structure in differentiated somatic S2 cells, suggesting that HP1α may be only required for the establishment but not for the maintenance of compartments at later stages [87]. Because peri-centromeric clustering and compartmentalization also occur in mammals, Zenk et al. infer that HP1 could have similar functions in mammalian embryos.

Additionally, Snf2h is the ATPase of the chromatin remodeling complex ISWI. In xenopus early embryos, the Snf2h knockdown can lead to severely weakened TADs and embryonic lethality (12 hpf, after ZGA) [21]. ISWI has recently been shown to mediate CTCF binding in mammalian cells [89], suggesting that the chromatin remodeling complex can play an essential role in establishing TAD structures, possibly through mediating CTCF binding.

### 4.5. Histone Modifications

During the embryonic development in mammals, histone modifications on the chromatin play critical roles in regulating the expression of developmental genes [90]. Recently, some studies have revealed a strong association between 3D chromatin structures and histone modifications [19,25,26,91,92]. For instance, A compartments are enriched for active histone modifications including H3K27ac and H3K4me3/me1, while B compartments contain the heterochromatin mark H3K9me37 in human lymphoblastoid cells [91]. Additionally, active chromatin marks H3K4me3 and H3K27ac are also significantly enriched at TAD boundaries [26]. There are increasing evidences that histone modifications are involved in regulating the 3D chromatin structure reprogramming in many species. SGC-CBP30, which is a bromodomain inhibitor of histone acetyltransferase Ep300a and Crebbp, can reduce H3K27ac modification levels. In zebrafish embryos, the inhibition of H3K27ac modification by SGC-CBP30 can lead to a reduction of insulation between a subset of putative super enhancers [19].

### 4.6. Phase Separation 

As cells proliferate during embryogenesis, basic anabolic metabolism and translational processes become more active. The nucleolus, an organelle involved in translation, becomes functionally mature from nucleolar precursor bodies (NPB) during embryogenesis [93,94]. Interestingly, it appears that shutting down ZGA and initiating nucleolus formation are not independent; rather, they are interconnected events. A recent study reported that the TRIM28/NCL/LINE1 complex can mediate both ZGA gene Dux repression and rRNA expression [95]. In mice, Dux is a 2C program transcription factor [96]. It is known that nucleolar integrity maintains the normal liquid–liquid phase separation (LLPS) of the nucleolus and the formation of peri-nucleolar heterochromatin (PNH). Upon defects in rRNA biogenesis, the natural state of nucleolus LLPS is disrupted, causing a dissociation of the NCL/TRIM28 complex from PNH [96]. The dissociation of the NCL/TRIM28 complex drives the 3D structure reorganization of PNH, which leads to the release of Dux from PNH to activate a 2C-like program [96]. These results indicate some kind of correlation between the normal nucleolar LLPS and the 3D chromatin structure within the nucleus. OCT4 is an important pioneer factor in pluripotent cells. A recent study firstly reported that during the pre-iPSC to PSC transition process, OCT4 can form liquid-like condensates and can regulate the TAD reorganization by OCT4-mediated phase separation [97].

Different from a conventional LLPS which is driven by protein–protein interactions, a recent study reveals that DNA–cohesin clusters also exhibit liquid-like behavior. This new form of phase separation named bridging-induced phase separation (BIPS) can use DNA–cohesin–DNA bridges as nucleation points for recruiting more cohesin complexes in vitro [98]. Therefore, the role for BIPS in chromatin organization in embryos would be interesting to explore further.

## 5. The Role of 3D Chromatin Structures in the Cellular Totipotency

**Figure 4:**
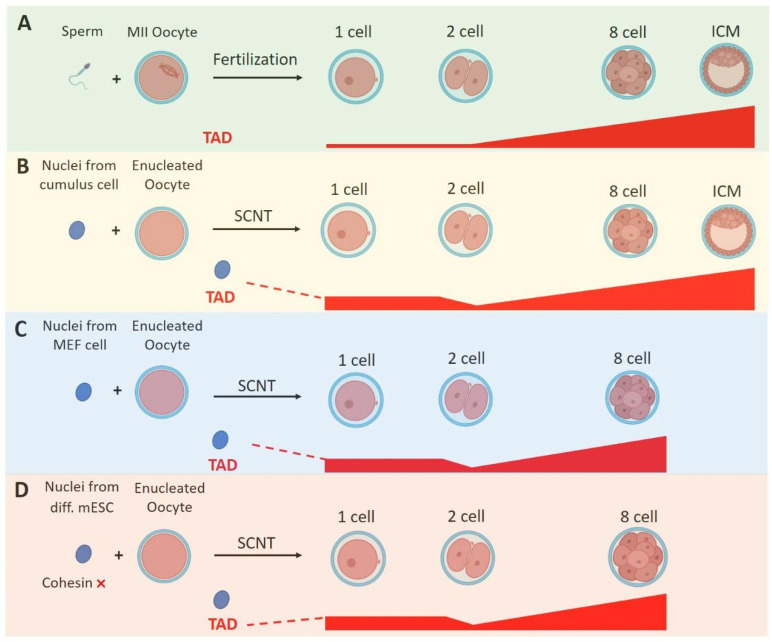
A schematic model comparing the reprogramming of chromatin organization in in vivo fertilized embryos and in vitro SCNT embryos. (**A**) The TAD dynamic during in vivo fertilized embryos development. (**B**) The TAD dynamics during SCNT embryo development. The SCNT embryos are derived from injecting the donor nuclei of terminally differentiated cumulus cells and enucleated oocytes. (**C**) The TAD dynamics in SCNT embryo development. The SCNT embryos derived from the donor nuclei of MEF cells and enucleated oocytes. (**D**) The TAD dynamics of SCNT embryos derived from cohesin-depleted donor nuclei.

It is noteworthy that the zygote is coupled with the acquisition of totipotency, namely the ability of a cell to generate all cell types including both the embryonic and extraembryonic tissues in an organism. It has been reported that the 3D chromatin structures in mouse totipotent zygotes and 2-cell embryos are remarkably relaxed compared with those in the later-stage embryos and somatic cells [23,26] (Figure 4A). Such relaxed chromatins within totipotent embryos are also observed in other species, including drosophila, zebrafish, xenopus and human [20,21,22,25,57]. Moreover, when a somatic nucleus is reprogramed to the totipotent state by somatic cell nuclear transfer (SCNT), its chromatin structure also becomes markedly relaxed [99,100] (Figure 4B,C). Recent studies indicate an interesting relationship between 3D chromatin structures and the totipotency in SCNT embryos [99,100]. Although they used different donor nuclei (MEF cell or cumulus), these two studies both reveal that during the SCNT embryo development, the transferred nucleus first enters a mitotic-like state (premature chromatin condensation) and then the SCNT embryos show stronger TADs than that in zygotes (Figure 4B,C). TADs in SCNT embryos become weaker at the 2-cell stage, followed by gradual consolidation (Figure 4B,C). Meanwhile, A/B compartments are markedly weakened in 1-cell SCNT embryos and become increasingly strengthened afterwards [99,100]. 

Moreover, there are relatively strong interactions between a key minor ZGA gene named Zscan4 and a nearby super enhancer cluster (2 Mb away) in fertilized-derived 2-cell embryos [99]. Such interactions become much weaker in SCNT early 2-cell embryos, which is consistent with the persisting TADs in SCNT embryos and the insufficient activation of Zscan4 [99,100]. Importantly, the depletion of cohesin in mESCs leads to the activation of minor ZGA genes, including Zscan4. The cohesin pre-depletion in the donor cells can also unsuppress genes in SCNT embryos and further improve SCNT efficiency in mouse embryos [100] (Figure 4D). However, according to the ChIP-seq, cohesin does not directly bind to Zscan4 according to the ChIP-seq data in mESCs. Therefore, it is proposed that the loss of TADs in fertilized embryos can alleviate TAD-mediated insulation and then release the super enhancer to activate Zscan4.

Embryonic stem cells (ESCs) are derived from inner cell mass (ICM). In contrast to zygotes, ESCs are considered to have developmental pluripotency because they can differentiate into all three embryonic germ layers but cannot differentiate into extraembryonic tissues. Intriguingly, in mouse ESC cultures, there exists a rare dynamic subset of cells which have the ability to develop into to both embryonic and extraembryonic tissues, showing a totipotent-like developmental potency similar to the 2-cell blastomeres [101]. These cells highly express 2-cell-specific transcripts, and are thus known as 2C-like cells (2CLCs). In a recent study, during the transition from ESCs to 2CLCs, although the A/B compartment patterns were largely maintained, the compartmentalization strength significantly decreased [102]. Additionally, there exists a global reduction of TAD insulation in 2CLCs compared with ESCs [102]. In 2CLCs, a set of pluripotent genes are downregulated in 2CLCs, such as Pou5f1, Sox2, Nanog, Myc, Klf4, Esrrb, Lin28a, and Rex1. This process is accompanied by reduced enhancer–promoter interactions [102]. On the other hand, the knockdown of key chromatin structure proteins, such as Ctcf, Smc1a, Smc3, and Rad21, can significantly increase the fraction of 2CLCs and upregulate 2C-specific genes, including Dux, Zscan4d, Zfp352, and Tdpoz4. This shows an enhanced ESC to 2CLC transition [102].

Together, these results shows that the relaxed chromatin structure is a unique feature of the totipotent cell, including in vivo totipotent embryos, in vitro SCNT embryos and 2CLCs. 

## 6. Conclusions and Perspectives

The early embryonic development in animals is a unique process during which the terminally differentiated gametes fuse and form into totipotent embryos. Eventually, the totipotent embryos can differentiate into a variety of cells, tissues, and organs. The ZGA is an unarguably important developmental event during the animal embryonic development. The coincidence of TAD establishment and the ZGA event in early embryos raises the question of why the timing of TAD establishment often coincides with ZGA in many species. The biological insights for the conservation of 3D genome reorganization in embryos needs to be further explored. 

Although Hi-C and its derived methods significantly advance our knowledge of chromatin structures in gametes and embryos, it still requires new methods to analyze the 3D genome in early embryos at fine resolutions using scarce material. GAM (genome architecture mapping) is a ligation-free method based on nuclear cryosections, which can map chromatin structures and detect high-complexity chromatin contacts [103]. IGS (in situ genome sequencing) simultaneously unifies the sequencing and imaging of DNA sequences, and can then spatially localize thousands of genomic loci to determine the chromatin organization at single-nuclear level [104]. These methods can detect long-range contacts that involve three or more DNA fragments, and can then resolve detailed chromatin organization within 3D nuclear space. Furthermore, SPRITE (split-pool recognition of interactions by tag extension) can simultaneously map DNA and RNA at genome loci and even incorporate protein localization, and can then generate combinatorial and spatial multi-way maps of DNA, RNA, and/or protein [105,106]. With the help of these advanced technologies, we can further unveil the chromatin structure reprogramming during embryogenesis in unprecedented detail.

Moreover, many mechanisms, such as insulator proteins, transcription factors and the phase separation described above, are evidenced to be involved in the regulation of chromatin structure reorganization during embryo development. As dictated by polymer physics [107], the combinatorial action and multilateral interplay of different architectural proteins, transcription factors, and histone modifications may modulate the chromatin polymer characteristic and change the thermodynamics of polymer phase separation to shape the 3D genome architecture. Therefore, the expectation of further investigations is to elucidate the multi-way molecular mechanisms for the 3D chromatin structures organization in embryos, which could then show promise toward fully unveiling the relationship of 3D genomic structure and cell totipotency.

## Figures and Tables

**Table 1:** Insulator and non-insulator proteins in domain formation. “D” for drosophila; “Z” for zebrafish; “X” for xenopus; “M” for mouse; “H” for human.

Gene	Species	Functions	References
CTCF	Z,X,M,H	function as an insulator protein in TAD establishment	[21,22,69,70]
Cohesin	D,M,H	function as a driver to extrude a chromatin loop	[80,81,82,85]
BEAF-32	D	BEAF-32 binds to specific DNA sequences, mediate long-range chromosomal contacts	[83,84]
Cp190	D	bind to BEAF-32, mediate long-range chromosomal contacts	[83,84]
Chromator	D	bind to BEAF-32, mediate long-range chromosomal contacts	[83,84]
Zelda	D	may relax local chromatin environment to help TAD boundaries and multi-way interaction formation	[25]
GAF	D	associate with Zelda to open chromatin	[86]
HP1α	D	bind to H3K9me3 in constitutive heterochromatin to establish B compartments	[87]
Snf2h	X	mediate CTCF binding to DNA for TAD establishment	[21]

## Data Availability

Not applicable.
